# α-Terpineol reverses *mcr*-mediated colistin resistance and potentiates colistin’s antibacterial activity in multidrug-resistant *Escherichia coli*

**DOI:** 10.3389/fcimb.2026.1908346

**Published:** 2026-08-26

**Authors:** Yujuan Li, Yi Yang, Ting Zhou, Jing Zhou, Jie Hu, Shuangshuang Liang, Zhenhua Xie, Qing Cheng, Yachao Jiang, Chao Jiang, Qing Liu

**Affiliations:** 1 College of Basic Medicine, Xiangnan University, Chenzhou, China; 2 Hunan Provincial Key Laboratory of Gut Microbiota and Chronic Disease Intervention, Chenzhou, China; 3 College of Clinical, Xiangnan University, Chenzhou, China; 4 College of Pharmacy, Xiangnan University, Chenzhou, China

**Keywords:** antibiotic adjuvant, colistin, *Escherichia coli*, MCR, multidrug-resistant, α-terpineol

## Abstract

Amid the growing antibiotic resistance crisis, colistin remains a last-line therapeutic option for multidrug-resistant (MDR) Gram-negative bacterial infections. However, the emergence and rapid dissemination of plasmid-mediated colistin resistance gene (*mcr*) have markedly reduced its clinical effectiveness. Utilizing colistin adjuvants to restore its antibacterial potency is a promising strategy to combat this threat. In this study, we aimed to investigate the synergistic effects of α-terpineol in combination with colistin against colistin-resistant MDR *Escherichia coli* (*E. coli*) and to elucidate the underlying mechanisms of this synergy. *In vitro* synergistic activity was evaluated using checkerboard microdilution assays, time-kill curve analyses, and resistance development studies. Mechanistic insights were obtained through transcriptomic analysis and fluorescence-probe based assays. *In vivo* efficacy was validated using *Galleria mellonella* and mouse acute peritonitis infection models. The results showed that the combination of α-terpineol and colistin exhibited synergistic bactericidal activity and suppressed the development of colistin resistance. α-Terpineol increased inner- and outer-membrane fluidity in *E. coli*, thereby enhancing colistin uptake. Furthermore, it inhibited superoxide dismutase (SOD) activity, preventing the conversion of O_2_^·–^ to H_2_O_2_ and causing superoxide accumulation. Elevated O_2_^·–^ levels damaged iron–sulfur clusters and impaired respiratory function. Together with the proton motive force (PMF) dissipation, these effects severely impaired ATP synthesis, ultimately sensitizing MDR *E. coli* to colistin. The combination also showed significant therapeutic efficacy in animal models. Our findings identify α-terpineol as a promising colistin adjuvant that restores antibacterial activity by disrupting membrane integrity and impairing antioxidant defenses, offering a viable strategy for treating MDR Gram-negative bacterial infections.

## 1 Introduction

Since the introduction of sulphonamides as the first effective antimicrobial agents, antibiotics have been used clinically for nearly a century (Davies and Davies, 2010; Liu et al., 2020). However, widespread antibiotic use has accelerated the emergence and global spread of antimicrobial resistance (AMR). Specifically, the proliferation of multidrug-resistant (MDR) infections, in which pathogens acquire resistance to multiple antibiotic classes, severely limits therapeutic options and increases the risk of treatment failure, posing a major clinical challenge (Alara and Alara, 2024). The AMR crisis threatens modern medicine by rendering previously treatable bacterial infections potentially life-threatening again (Kuehn, 2020). As AMR evolves and spreads rapidly, conventional antibiotics are nearly exhausted, while the development of novel antibacterial agents remains limited. Consequently, innovative strategies are essential to bridge the gap between the slow pace of therapeutic development and the growing AMR threat.

Colistin, a cationic polypeptide antibiotic, targets lipopolysaccharide (LPS) of the outer membrane of Gram-negative bacteria, causing membrane lysis and cell death (Yu et al., 2015). Colistin serves as a vital last-line agent for MDR Gram-negative bacterial infections (Liu et al., 2019). However, the global spread of transferable plasmid-mediated colistin resistance gene (*mcr*), encoding MCR enzymes, severely compromised its clinical efficacy (Sun et al., 2018). These genes encode MCR enzymes, which confer colistin resistance by adding a positively charged phosphoethanolamine to lipid A, thereby reducing the net negative charge of LPS and impairing colistin binding (MacNair et al., 2018; Hussein et al., 2021), posing a serious challenge in the treatment of infectious diseases (Yu et al., 2015; Rhouma et al., 2016). Colistin resistance is increasingly observed in clinically important opportunistic pathogens, particularly *Escherichia coli* (*E. coli*) (Xie et al., 2022); this trend underscores the urgent need to develop resistance-reversing agents or antibacterial adjuvants.

Combination therapeutic strategies based on natural products offer a promising approach to address antibiotic resistance (Xiao et al., 2023). Plant-derived bioactive compounds, owing to their multi-target properties, produce synergistic antibacterial effects combined with conventional antibiotics, demonstrating unique advantages and potential in combating bacterial resistance (Zhong et al., 2023; Wang et al., 2024). In this study, we evaluated the synergistic potential of α-terpineol (2-(4-methylcyclohex-3-en-1-yl) propan-2-ol) in combination with colistin against *mcr*-positive bacteria. Our results demonstrated that this combination exhibited strong synergistic effects, highlighting its considerable potential as a colistin adjuvant. However, systematic investigation into the role of α-terpineol in reversing or reducing bacterial resistance remains limited. Therefore, this study established an α-terpineol–colistin combination therapy system and evaluated its synergistic antibacterial mechanisms and pharmacodynamics against colistin-resistant MDR *E. coli*. Our findings may support the development of adjuvant-based strategies to restore antibiotic efficacy against drug-resistant Gram-negative pathogens.

## 2 Materials and methods

### 2.1 Bacterial strains and chemical reagents

This study included *mcr*-positive MDR *E. coli* strains with previously characterized resistance profiles and whole-genome sequencing data. The resistance backgrounds of these strains, which included three *mcr*-1 positive strains (LN175, AG210, AG227), one *mcr*-3 positive strain (22A13), one *mcr*-9 positive strain (25N30), and one *mcr*-10 positive strain (25A20), are summarized in **Supplementary Figure 1**. Unless otherwise stated, all strains were inoculated onto Mueller–Hinton agar (MHA) containing 4 μg/mL colistin to achieve resistance phenotypes, and colonies were transferred and grown in Mueller–Hinton broth (MHB) for further experiments. All the media were purchased from Landbridge Co., Ltd. (Beijing, China). α-Terpineol, colistin, and the fluorescence probes 2′,7′-bis-(2-carboxyethyl)-5-(and-6)-carboxyfluorescein acetoxymethyl ester (BCECF-AM), N-phenyl-1-naphthylamine (NPN), and 3,3′-dipropylthiadicarbocyanine iodide (DiSC_3_(5)) were all purchased from Aladdin Biochemical Technology Co., Ltd. (Shanghai, China). Propidium iodide (PI) and 2′,7′-dichlorodihydrofluorescein diacetate (H_2_DCF-DA) were purchased from Biosharp Biotechnology Co., Ltd. (Beijing, China). The enhanced ATP assay kit and Cell Counting Kit-8 were obtained from Dalian Meilun Biotechnology Co., Ltd. (Dalian, China). The Unizol total RNA extraction reagent kit was purchased from Genes and Biotechnology Co., Ltd. (Beijing, China). Dihydroethidium was sourced from Beyotime Biotechnology (Shanghai, China). Cell ferrous iron colorimetric assay kit was obtained from Elabscience Biotechnology Co., Ltd. (Wuhan, China). The Hydrogen Peroxide (H_2_O_2_) Content assay kit, superoxide dismutase (SOD) activity assay kit and hydroxyphenyl fluorescein (HPF) were supplied by Beijing Solarbio Science & Technology Co, Ltd. (Beijing, China).

### 2.2 Checkerboard assay

Synergistic interactions between antibiotics and α-terpineol were assessed using a checkerboard microdilution method in a 6 × 6 matrix format. According to their respective minimum inhibitory concentrations (MICs), α-Terpineol and colistin were serially diluted to establish six concentration gradients (0, 1/16× MIC, 1/8× MIC, 1/4× MIC, 1/2× MIC, and 1× MIC). Overnight cultures of *mcr*-positive MDR *E. coli* strains were adjusted to 0.5 McFarland standard and diluted 1:1000 in fresh MHB to obtain a final inoculum of approximately 5 × 10^5^ CFU/mL. The 50 μL of each drug at the corresponding concentration and 100 μL of bacterial suspension were added to each well of a 96-well microtiter plate, achieving the designated final concentrations per well. Following a 24 h-incubation at 37 °C, the optical density (OD) was measured at 600 nm. The fractional inhibitory concentration index (FICI) was calculated as follows:


$$\text{FICI}={\text{FICI}}_{a}+{\text{FICI}}_{b}=(\frac{{\text{MIC}}_{ab}}{{\text{MIC}}_{a}})+(\frac{{\text{MIC}}_{ba}}{{\text{MIC}}_{b}})$$

where MIC*_a_* and MIC*_b_* represent the MICs of α-terpineol and colistin alone, respectively, whereas MIC*_ab_* and MIC*_ba_* denote their MICs in combination. Interpretation of FICI values was based on established criteria: synergism (FICI ≤ 0.5), additive effect (0.5 < FICI ≤ 1), indifferent effect (1 < FICI ≤ 2), and antagonism (FICI > 2).

### 2.3 Time-dependent killing assay

*E. coli* LN175 was cultured overnight for 12 h to obtain a bacterial suspension, diluted to approximately 10^4^ CFU/mL in fresh MHB, and treated with subinhibitory concentrations of colistin (8 μg/mL), α-terpineol (0.2 mg/mL), or their combination. At 0, 3, 6, 9, and 24 h, 100 μL aliquots from each treatment were collected and serially diluted. The dilutions were spotted onto MHA, and colony counts were determined after overnight incubation at 37 °C.

### 2.4 Resistance development study

The bacterial inoculum was prepared and processed following the same procedure as described in the time-kill curve assay above. Subsequently, this suspension was incubated in MHB containing sublethal concentrations of colistin at 37 °C, with or without sublethal α-terpineol. The MIC values of each group were determined every 24 h, and the culture medium and drugs were replaced accordingly. The passage was continued for 28 days, with drug concentrations adjusted according to observed MIC values.

### 2.5 Transcriptomic analysis

Transcriptomic analysis was performed in *E. coli* LN175 cells, which were cultured in MHB to the exponential growth phase and then treated for 1 h with colistin (8 μg/mL) alone or in combination with α-terpineol (0.2 mg/mL). Bacterial cells were harvested via centrifugation at 5,000 × *g* for 5 min at 4 °C. Total RNA was extracted and quantified by Novogene Biotechnology Co., Ltd. (Beijing, China). Following quality control, libraries were pooled based on the effective concentration and the required sequencing depth, and subjected to Illumina sequencing. Raw sequencing reads were filtered for quality and aligned to the *E. coli* LN175 reference genome. Gene expression levels were quantified using the fragments per kilobase of transcript per million mapped reads method. Differentially expressed genes (DEGs) were identified by applying the criteria of BH-adjusted *P*-values (Padj) ≤ 0.05 and fold change (FC) ≥ 2 (∣log_2_ FC∣ ≥ 1). Differential expression analysis between the two groups was performed using the NovoMagic Cloud Platform (http://magic.novogene.com/) provided by Novogene Bioinformatics Technology Co., Ltd. (Beijing, China).

### 2.6 Scanning electron microscopy

*E. coli* LN175 cells were treated with the designated concentrations of the test agents, the bacterial suspensions were incubated at 37 °C for 1 h. The cells were then centrifuged at 4,000 × *g* for 5 min, and the supernatant was discarded. The bacterial pellets were washed with phosphate-buffered saline (PBS), fixed with 2.5% glutaraldehyde, dehydrated, and dried. Subsequently, the samples were attached to conductive carbon double-sided tape placed on the sample stage of an ion sputter coater and sputter-coated with gold for 30 s. Morphological changes of the bacteria were observed under a scanning electron microscope (SEM).

### 2.7 Biochemical parameters assay

*E. coli* LN175 was used as the indicator strain. Overnight cultures of *E. coli* LN175 were diluted 1:100 into fresh MHB and incubated for 6 h to reach the exponential growth phase. The bacterial density was then adjusted to 0.5 McFarland standard. Tests using reagent kits were performed according to the manufacturer’s instructions. Unless otherwise specified, in fluorescent-probe experiments, the bacterial suspension was adjusted to the desired turbidity using PBS, incubated with the corresponding probe at 37°C in the dark for 30 min, and then 190 μL of the probe-containing suspension was dispensed into each well of a black 96-well plate. Subsequently, 10 μL of α-terpineol and colistin at different concentrations were added to the respective wells to achieve the final concentrations designated for each experimental group. The plate was further incubated at 37 °C in the dark for 1 h and fluorescence intensity was measured for all fluorescence-probe experiments using a SpectraMax M5e microplate reader (Molecular Devices, USA) after treatment. Unless otherwise specified, the experimental groups included a control group, a colistin group (1/4 × MIC), α-terpineol groups (1/8 × MIC, 1/4 × MIC, 1/2 × MIC), and combination groups of colistin (1/4 × MIC) with α-terpineol (1/8 × MIC, 1/4 × MIC, 1/2 × MIC).

#### 2.7.1 Biofilm formation inhibition assay

Bacterial suspension and the designated concentrations of drugs were added to 96-well plates. After 24 h incubation, the non-adherent planktonic bacteria were discarded, and the remaining adherent biofilm was fixed with fixative for 1 h, washed with PBS, and stained with crystal violet (0.05%) (Xilong Scientific Co., Ltd., China) for 30 min. Following another washing step, 95% ethanol was added to dissolve the bound dye. The absorbance of each well was measured at 595 nm using a multi-mode microplate reader (Yang et al., 2023), and the biofilm inhibition rate was calculated.

#### 2.7.2 Membrane fluidity assay

The bacterial suspensions were adjusted to the target turbidity using 4-(2-hydroxyethyl)-1-piperazineethanesulfonic acid (HEPES), and incubated with Laurdan (10 μM; Macklin, Shanghai, China). The following treatment groups were established: a blank control, colistin group (8 μg/mL), α-terpineol group (0.2 mg/mL), and combination group of α-terpineol. The excitation wavelength was 350 nm, and the emission wavelengths were 440 and 490 nm. Generalized polarization (GP) value was calculated using the following equation:


GP=(I440−I490)/(I440+I490)

#### 2.7.3 Outer membrane permeability assay

NPN fluorescent probe was employed at a final concentration of 10 μM. Following incubation, fluorescence intensity was measured using a microplate reader (excitation wavelength: 350 nm, emission wavelength: 420 nm).

#### 2.7.4 Cell membrane integrity assay

The fluorescent probe PI was applied at a concentration of 10 μM during incubation. Fluorescence signals were subsequently measured using a microplate reader (excitation wavelength: 535 nm, emission wavelength: 615 nm).

#### 2.7.5 Transmembrane proton concentration

The bacterial suspension was adjusted to the target turbidity using HEPES. The concentration of the probe BCECF-AM was set at 5 μM. Fluorescent intensity changes were monitored (excitation wavelength: 520 nm, emission wavelength: 535 nm).

#### 2.7.6 Membrane depolarization assay

The bacterial suspension was adjusted to the target turbidity using HEPES. The concentration of the probe DiSC_3_(5) was set at 0.5 μM. Fluorescence was measured using a microplate reader (excitation wavelength: 622 nm, emission wavelength: 670 nm).

#### 2.7.7 Measurement of intracellular ATP content

The intracellular ATP content of *E. coli* LN175 was quantified using a commercial assay kit. Following 1 h of drug treatment, the bacterial suspension was centrifuged at 5,000 × *g* for 5 min and the supernatant was discarded. The bacterial pellet was lysed with lysis buffer at 4 °C for 1 h, followed by centrifugation at 10,000 × *g* for 5 min. A 10-μL aliquot of the resulting supernatant was then transferred into a black 96-well microplate containing 100 μL of ATP detection reagent. The ATP levels were then evaluated based on luminescence signals.

#### 2.7.8 Measurement of total reactive oxygen species levels

Intracellular ROS levels were measured using a commercial assay kit. Following the manufacturer’s instructions, bacterial samples were first treated with the respective drugs, followed by incubation with the ROS-sensitive fluorescent probe at 37 °C in the dark for 30 min. After incubation, the samples were centrifuged, supernatant was removed, and bacterial pellets were resuspended in PBS. Fluorescence intensity was measured using a microplate reader (excitation wavelength: 488 nm, emission wavelength: 525 nm).

#### 2.7.9 Measurement of superoxide anion levels

O_2_^·–^ levels were determined using 10 μM dihydroethidium as the fluorescent probe. Fluorescence was measured using a microplate reader (excitation wavelength: 535 nm, emission wavelength: 610 nm).

#### 2.7.10 Measurement of hydrogen peroxide levels

A hydrogen peroxide assay kit was used to detect intracellular hydrogen peroxide levels. The absorbance of each well was measured at 415 nm using a microplate reader.

#### 2.7.11 Measurement of hydroxyl radical levels

The HO^·^ level was measured using 5 μM HPF as the fluorescent probe. The probe was incubated with the sample for 15 min. Fluorescence was measured using a microplate reader (excitation wavelength: 490 nm, emission wavelength: 515 nm).

#### 2.7.12 Ferrous iron content determination assay

Bacterial cultures were harvested after co-incubation with α-terpineol, colistin, or their combination and homogenized via sonication. Cell lysates were collected, and intracellular iron content was determined using the Fe^2+^ iron colorimetric assay kit (Elabscience Biotechnology Co., Ltd., Wuhan, China).

#### 2.7.13 Molecular docking and molecular dynamics simulation

Molecular docking was performed to predict the binding mode and affinity of α-terpineol toward manganese superoxide dismutase (Mn-SOD). The *E. coli* Mn-SOD crystal structure (PDB ID: 1IX9, 0.90 Å) was obtained from RCSB PDB, and α-terpineol (PubChem CID: 443162) from PubChem. The biological assembly (homodimer, chains A and B) was used for docking. Receptor and ligand were prepared with AutoDock Tools 1.5.7: water molecules were removed, non-polar hydrogens were merged, Gasteiger charges were assigned, and PDBQT files were generated. Ligand rotatable bonds were auto-detected, and the receptor was kept rigid. Docking was performed using AutoDock Vina 1.2.7, with the binding site centered on the manganese ion and surrounding conserved residues. The pose with the lowest binding energy was selected for subsequent analysis. Visualization was performed with PyMOL, and 2D interaction diagrams were used to characterize hydrogen bonds and hydrophobic contacts. MD simulation was performed using GROMACS 2022.3 (Abraham et al., 2015; Spoel et al., n.d). The protein and ligand were parameterized with the Amber99sb-ildn and GAFF force fields, respectively; ligand RESP charges were derived via AmberTools22 with Gaussian 16W and incorporated into the topology. The system was solvated with TIP3P water, neutralized with Na^+^, and subjected to periodic boundary conditions. After steepest descent energy minimization, 50,000-step (100 ps) NVT and NPT equilibrations were performed (τT = 0.1 ps). The production run lasted 100 ns (2 fs time step) with LINCS bond constraints and PME electrostatics. Trajectory analyses included Root-mean-square deviation (RMSD), Root-mean-square fluctuation (RMSF), radius of gyration (Rg), solvent-accessible surface area (SASA), and hydrogen bond counts. Binding free energy and per-residue decomposition were calculated via MM/GBSA (without entropy term), and the free energy landscape was generated using the gmx sham module.

#### 2.7.14 SOD activity assay

SOD activity was determined using a commercial assay kit. After 1 h of drug treatment, the bacterial suspension was centrifuged at 5000 × *g* for 5 min and the supernatant was removed. Subsequent sample processing and reaction setup were performed strictly according to the manufacturer’s instructions. Following mixing, the samples were incubated at 37 °C for 30 min, and the absorbance was measured at a wavelength of 560 nm.

#### 2.7.15 ROS rescue assays

These experiments were conducted according to the checkerboard dilution method described above. To eliminate the accumulation of O_2_^·–^, ferric chloride (FeCl_3_, 250 μmol/L) was added to the culture medium. Furthermore, to eliminate the effects of ROS on resistant bacteria, vitamin C (10 mmol/L) was added separately. After overnight incubation at 37 °C, the absorbance of bacterial cultures was measured using a microplate reader at 600 nm for both assays.

### 2.8 Animal studies

For both *Galleria mellonella* and mouse infection models, the bacterial inoculum was optimized in preliminary dose-finding experiments. The lowest dose that achieved 80–100% mortality in untreated controls within the observation period (5 days for *G. mellonella* larvae and 7 days for mice) was selected for formal therapeutic evaluations. Mortality in the untreated groups during the formal experiments confirmed successful infection. Colistin doses were calculated based on the standard animal dose conversion formula from human-equivalent doses (8 mg/kg for mice and 30 mg/kg for larvae), consistent with previously reported doses (7.5–10 mg/kg) (MacNair et al., 2018; Liu et al., 2020). α-Terpineol doses (30 mg/kg for mice and 200 mg/kg for larvae) were determined based on preliminary LD_50_ data, selecting concentrations below the lethal threshold, followed by dose-finding experiments within this safe range to identify optimal synergistic doses. Animals were randomly assigned to groups using a random number table, with the investigator blinded to allocation during administration and monitoring; sample sizes (n = 16 for larvae, n = 10 for mice) were determined by power analysis, and mice of both sexes (1:1) were included.

#### 2.8.1 *Galleria mellonella* infection model

*G. mellonella* larvae were randomly assigned to four experimental groups (n = 16 per group). Each larva was infected via injection of 10 μL of an *E. coli* LN175 suspension (3.0 × 10^8^ CFU/larvae) into the left posterior proleg. One hour post-infection, *G. mellonella* larvae were injected with normal saline, colistin (30 mg/kg), α-terpineol (200 mg/kg), or the combination of colistin (30 mg/kg) and α-terpineol (200 mg/kg) at the same position. *G. mellonella* larvae were monitored and recorded daily over a 5-day period, and survival was recorded.

#### 2.8.2 Mouse peritonitis–sepsis infection model

Specific pathogen-free Kunming mice (weight 18–22 g, male: female = 1:1) were stratified by sex and body weight, then randomly assigned via a computer-generated sequence to five groups (n = 10 per group, half male and half female in each group) and intraperitoneally inoculated with *E. coli* LN175 (200 μL, 3.0 × 10^8^ CFU/mouse). One hour post-infection, mice received a single intraperitoneal dose of normal saline, colistin (8 mg/kg), α-terpineol (30 mg/kg), or colistin (8 mg/kg) combined with α-terpineol (15 or 30 mg/kg). Survival was monitored daily for 7 days by an observer blinded to group allocation.

### 2.9 Statistical analysis

Experimental data were statistically analyzed using GraphPad Prism software (version 9.5, San Diego, CA, USA). All experiments were performed with at least three independent biological replicates, and data are presented as mean ± standard deviation. Differences between groups were conducted using one-way analysis of variance. Statistical significance was defined as follows: ^*^P < 0.05, ^**^P < 0.01, ^***^P < 0.001.

## 3 Results

### 3.1 α-Terpineol potentiates the bactericidal activity of colistin and limits the emergence of resistance

**Figure 1:**
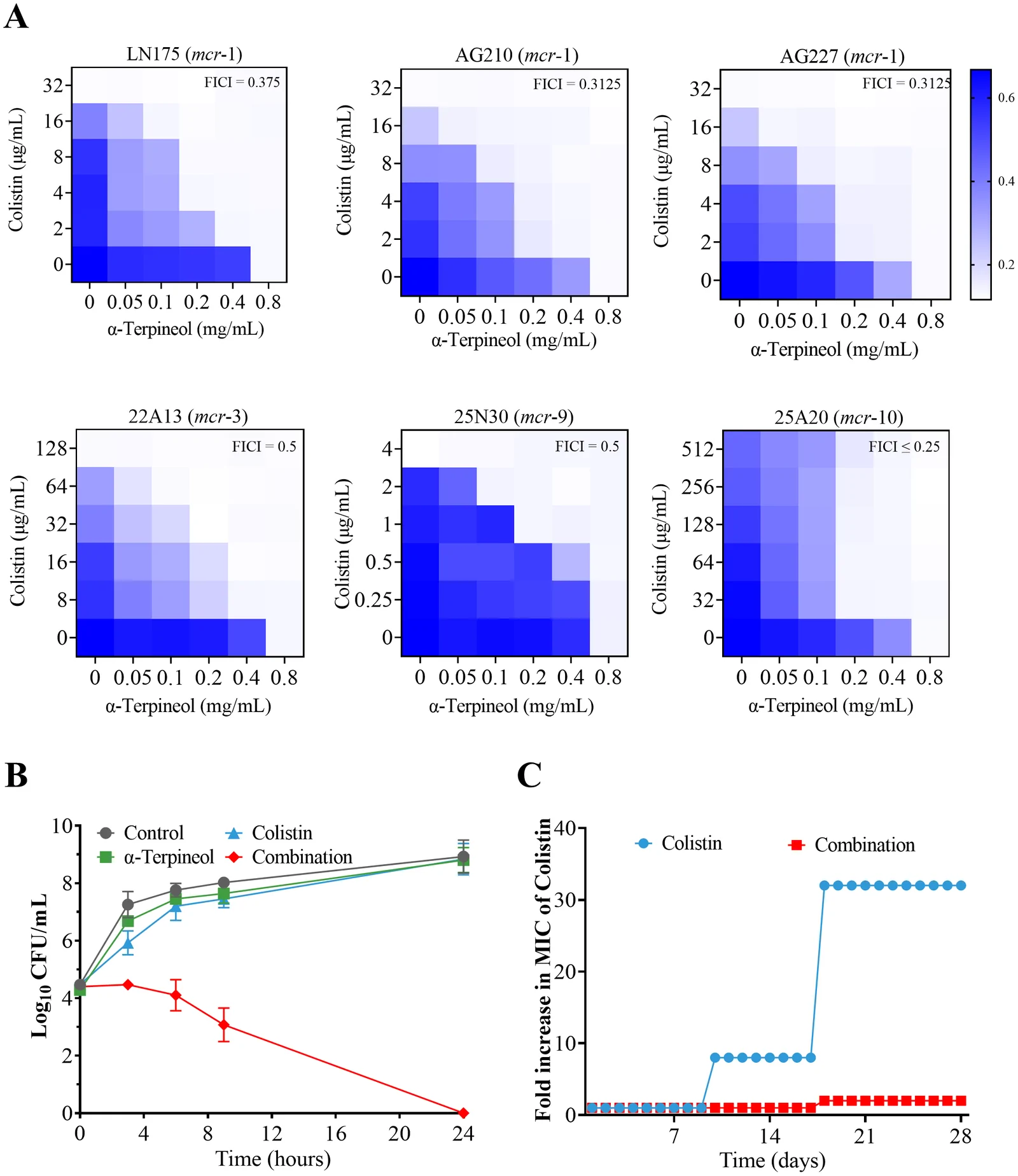
α-Terpineol restores colistin activity against *mcr*-positive MDR *E. coli* and reduces the development of colistin resistance. **(A)** Checkerboard broth microdilution assays. **(B)** Time-dependent killing curve. **(C)** Resistance development study.

MDR *E. coli* strains harboring different genotypes were used as model strains to investigate the potential synergistic antibacterial effect of α-terpineol combined with colistin against resistant bacteria using the checkerboard method. The results showed that the combination of α-terpineol and colistin exhibited significant synergistic activity against colistin-resistant MDR strains, with FICI values ≤ 0.5 (**Figure 1A**). These findings indicate that α-terpineol enhances the antibacterial activity of colistin against resistant strains. LN175 *E. coli* strain was selected as the representative among the tested *mcr*-positive isolates, based on its moderate colistin resistance (MIC = 32 μg/mL), carriage of the *mcr*-1 gene, and multidrug-resistant background. To further evaluate the synergistic bactericidal activity, time-kill curve analysis was performed on this strain during the exponential growth phase. Neither α-terpineol nor colistin alone was able to kill *E. coli* effectively. In contrast, the combination significantly reduced the bacterial load by approximately 4 log_10_ CFU/mL, confirming the synergistic bactericidal effect (**Figure 1B**). The effect of α-terpineol on the development of bacterial resistance was further assessed by serially passaging the standard strain ATCC 25922 in medium containing colistin with or without α-terpineol. After 28 days of continuous passage, colistin alone led to a 32-fold increase in the MIC, whereas in the presence of α-terpineol, the MIC increased only 2-fold (**Figure 1C**). The potential cytotoxicity of α-terpineol was evaluated using the L929 cells and 4% rabbit red blood cells. Within the tested concentration range, α-terpineol exhibited low cytotoxicity and hemolytic activity, indicating favorable biosafety (**Supplementary Figure 2**). These findings demonstrate that α-terpineol enhances the bactericidal activity of colistin against resistant *E. coli* and minimizes the emergence of resistance.

### 3.2 α-Terpineol disrupts membrane homeostasis and impairs the energy system to assist colistin in triggering oxidative stress

**Figure 2:**
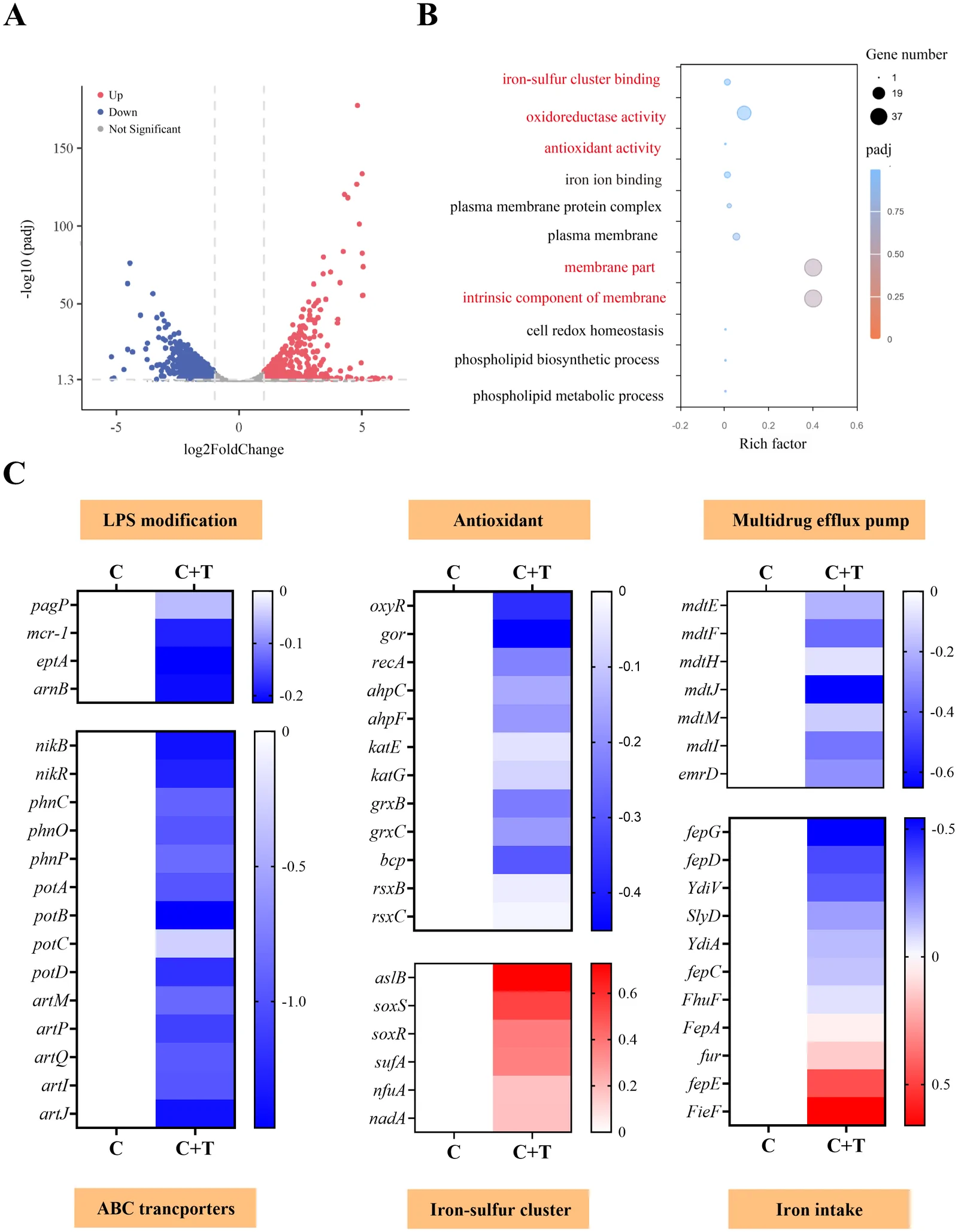
Transcriptomic analysis of *E. coli* treated with colistin or the combination of colistin and α-terpineol. **(A)** Volcano plot: the x- and y-axes represent expression changes and the corresponding degree of statistical significance, respectively. **(B)** Gene ontology enrichment analysis of differentially expressed genes (DEGs) in *E. coli* after exposure to colistin or the combination of colistin and α-terpineol. **(C)** Selected DEGs involved in lipopolysaccharide modification, antioxidant response, multidrug efflux pumps, ATP-binding cassette (ABC) transporters, iron–sulfur clusters, and iron uptake. C, colistin alone; C+T, the combination of colistin and α-terpineol.

To further explore the specific mechanism of α-terpineol’s synergistic antibacterial activity with colistin, we performed transcriptional analysis on colistin-resistant MDR *E. coli* treated with colistin alone or colistin plus α-terpineol. Compared to colistin treatment alone, the combined treatment resulted in the up-regulation of 559 genes and down-regulation of 772 genes (> two-fold) (**Figure 2A**). Gene Ontology (GO) enrichment analysis revealed that these DEGs were associated with pathways such as membrane part, intrinsic component of membrane, oxidoreductase activity, antioxidant activity, and iron–sulfur cluster binding (**Figure 2B**). Specifically, we observed upregulated expression of the genes involved in the iron–sulfur cluster functions, alongside downregulation of genes related to antioxidant responses, LPS modification, ATP-binding cassette (ABC) transporters and multidrug efflux pumps. Interestingly, we observed upregulation of genes that inhibit iron uptake, such as *fur*, alongside downregulation of genes that promote iron uptake. Both changes converge to the same outcome: bacterial iron uptake is arrested (**Figure 2C**). RT-qPCR analysis of representative gene expression profiles confirmed consistency with the transcriptomic results (**Supplementary Figure 3**). These findings indicate that α-terpineol exerts synergistic activity with colistin by modulating key physiological processes, including membrane-associated functions such as iron uptake, multidrug efflux pumps, and LPS modification, as well as antioxidant defense systems.

**Figure 3:**
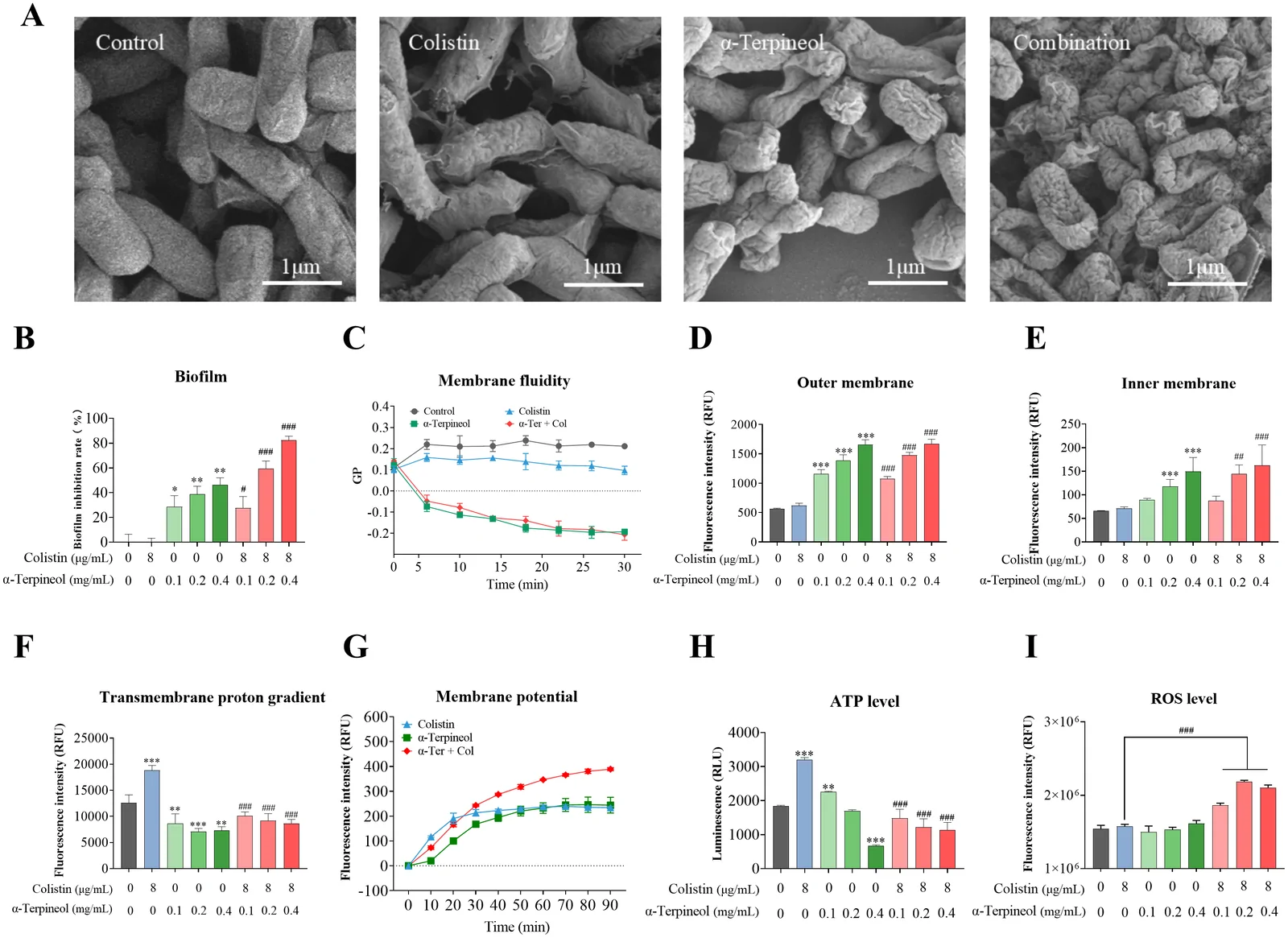
α-Terpineol disrupts membrane homeostasis and impairs the energy system to assist colistin in triggering oxidative stress. **(A)** Scanning electron microscopy **(SEM)** results. Scale bar, 1 μm. **(B)** Bacterial biofilm formation inhibition rate assay. **(C)** Membrane fluidity. **(D)** Outer membrane permeability test. **(E)** Inner membrane permeability test. **(F)** Transmembrane proton gradient test. **(G)** Membrane potential detection. **(H)** ATP content detection. **(I)** Total reactive oxygen species (ROS) detection. All experiments were performed with biological replicates and are presented as mean ± standard deviation. An unpaired one-way analysis of variance among multiple groups was used to calculate *P*-values (^*^P < 0.05, ^**^P < 0.01, ^***^P < 0.001 *vs* control; ^#^*P* < 0.05, ^##^*P* < 0.01, ^###^*P* < 0.001 *vs* colistin).

Given that GO analysis indicated α-terpineol significantly affected the expression of membrane- and LPS-modification-related genes, we used SEM to examine bacterial morphology under different treatments. *E. coli* treated with α-terpineol alone exhibited significant membrane shrinkage, which was further exacerbated upon combination with colistin, but no membrane perforation was observed (**Figure 3A**). Additionally, α-terpineol significantly inhibited biofilm formation (*P* < 0.05) (**Figure 3B**). Based on the phenotype of membrane shrinkage rather than disruption, we hypothesized that α-terpineol may compromise membrane structural support by increasing membrane fluidity (Zielińska et al., 2020), thereby leading to shrinkage. To verify this hypothesis, we employed specific fluorescent probes to detect membrane fluidity, outer membrane permeability, and inner membrane permeability. The results showed that α-terpineol treatment significantly increased lipid membrane fluidity (**Figure 3C**), leading to a significant increase in the permeability of both the inner and outer membranes (*P* < 0.001) (**Figures 3D, E**). Collectively, α-terpineol disrupts the normal homeostasis of membrane fluidity, impairs membrane permeability, and thus facilitates the entry of colistin into bacteria.

MDR Gram-negative bacteria commonly utilize active efflux mechanisms to extrude antibiotics out of the cell, thereby reducing the effective intracellular concentration of the antibiotic (Yang et al., 2023). Efflux pumps are energetically dependent on the proton motive force (PMF) of the membrane; disruption of PMF impairs ATP synthesis, thereby compromising efflux pump function (Bhargava and Collins, 2015; Sun et al., 2021). PMF serves as the core driving force of bacterial membrane energy metabolism and consists of two key components: transmembrane proton gradient and membrane potential (Yang et al., 2023). Therefore, we measured the transmembrane potential and proton gradient in bacteria under different treatments. The results showed that α-terpineol significantly dissipated the proton gradient (**Figure 3F**) and when combined with colistin, led to substantial dissipation of the membrane potential (**Figure 3G**). Given the close relationship between PMF and ATP synthesis, we further measured ATP levels. Notably, treatment of colistin-resistant MDR *E. coli* with sub-MIC concentrations of colistin induced a dramatic surge in intracellular ATP levels (*P* < 0.001) (**Figure 3H**), a phenomenon potentially associated with the activation of resistance genes and metabolic resuscitation mechanisms (Xu et al., 2017). In contrast, α-terpineol significantly inhibited the colistin-induced hyperactive energy metabolism (**Figure 3H**).

Although the bactericidal mechanism of colistin is not fully understood, it has been proposed that hydroxyl radical oxidative damage triggered by reactive oxygen species (ROS) is closely linked to colistin-induced rapid bacterial death (Sampson et al., 2012; Tan et al., 2021). Compared with the standard strain ATCC 25922 *E. coli*, colistin-resistant strains required higher concentrations of colistin to generate lethal levels of ROS (**Supplementary Figures 4A, B**), suggesting that *mcr*-mediated resistance effectively suppresses the hydroxyl-radical death pathway triggered by colistin. We further investigated whether α-terpineol could restore colistin-induced oxidative damage. To this end, we examined the effect of different concentrations of α-terpineol on colistin-induced ROS levels in the presence of a sub-inhibitory concentration of colistin (8 μg/mL). The results demonstrated that, compared with colistin or α-terpineol alone, the combination of α-terpineol and colistin significantly enhanced ROS production in a concentration-dependent manner (*P* < 0.001) (**Figure 3I**). Notably, sub-MIC concentrations of α-terpineol alone had no direct effect on total ROS levels (**Supplementary Figure 4C**). Collectively, these data indicate that α-terpineol weakens the resistance barrier, disrupts membrane homeostasis, inhibits membrane energy metabolism, and interferes with efflux pump function, thereby creating favorable conditions for colistin entry and the induction of ROS stress.

### 3.3 α-Terpineol suppresses SOD activity, disrupts the antioxidant system, and intensifies oxidative damage in colistin-resistant bacteria

**Figure 4:**
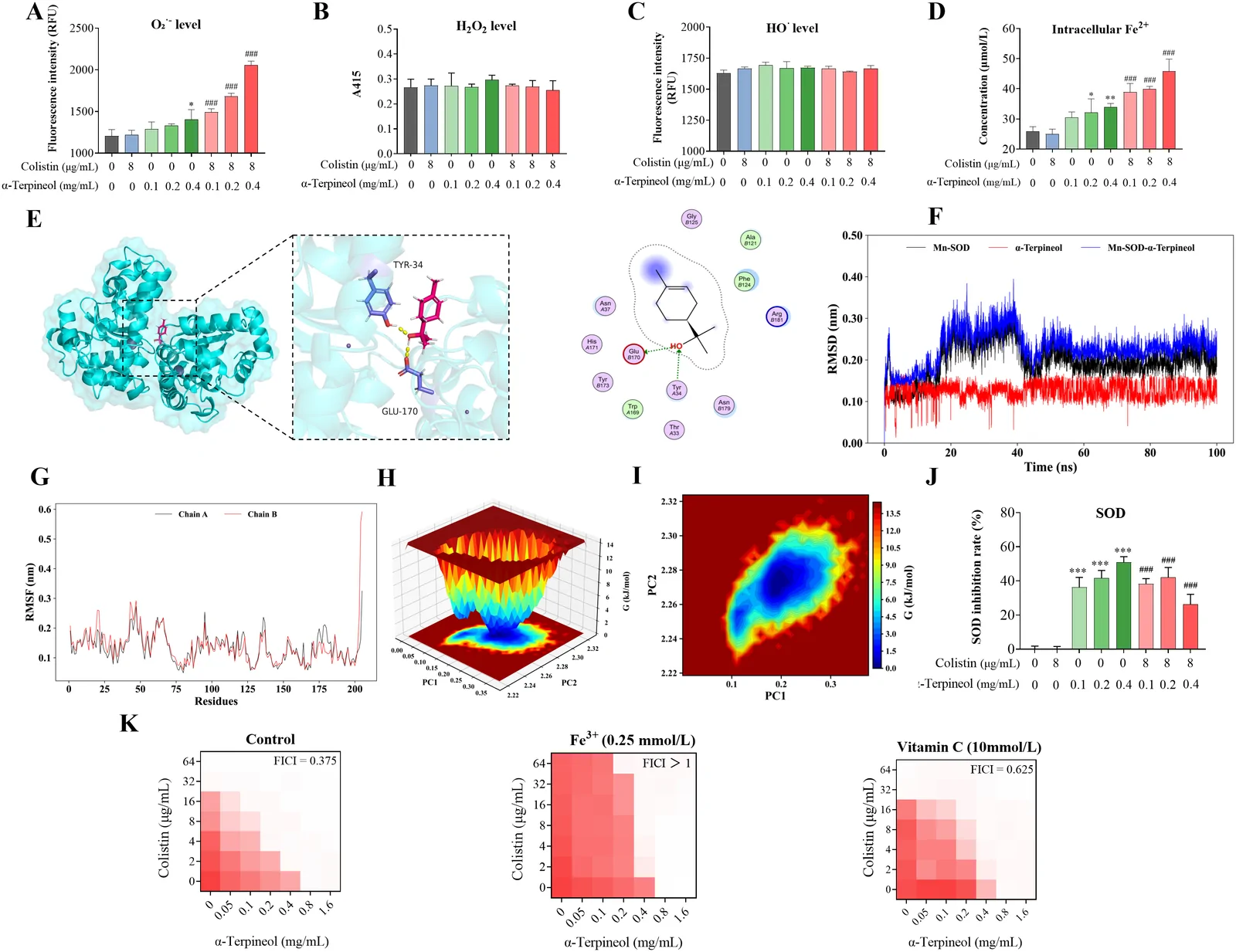
α-Terpineol suppresses superoxide dismutase (SOD) activity and disrupts the antioxidant system in colistin-resistant bacteria. **(A)** Intracellular O_2_^·–^ content. **(B)** H_2_O_2_ content. **(C)** HO^·^ content. **(D)** Ferrous ion (Fe^2+^) concentration. **(E)** Molecular docking of α-terpineol with Mn-SOD. **(F)** Root-mean-square deviation (RMSD). **(G)** Root-mean-square fluctuation (RMSF) of protein residues. **(H)** Three-dimensional Gibbs free energy landscape. **(I)** Two-dimensional Gibbs free energy landscape. **(J)** Intracellular SOD activity. **(K)** Effects of Fe^2+^ (0.25 mmol/L) and vitamin C (10 mmol/L) on the synergistic activity of α-terpineol and colistin. All experiments were performed with biological replicates and are presented as mean ± standard deviation. An unpaired one-way analysis of variance among multiple groups was used to calculate *P*-values (^**^P < 0.01, ^***^P < 0.001 *vs* control; ^#^*P* < 0.05, ^##^*P* < 0.01, ^###^*P* < 0.001 *vs* colistin).

**Figure 5:**
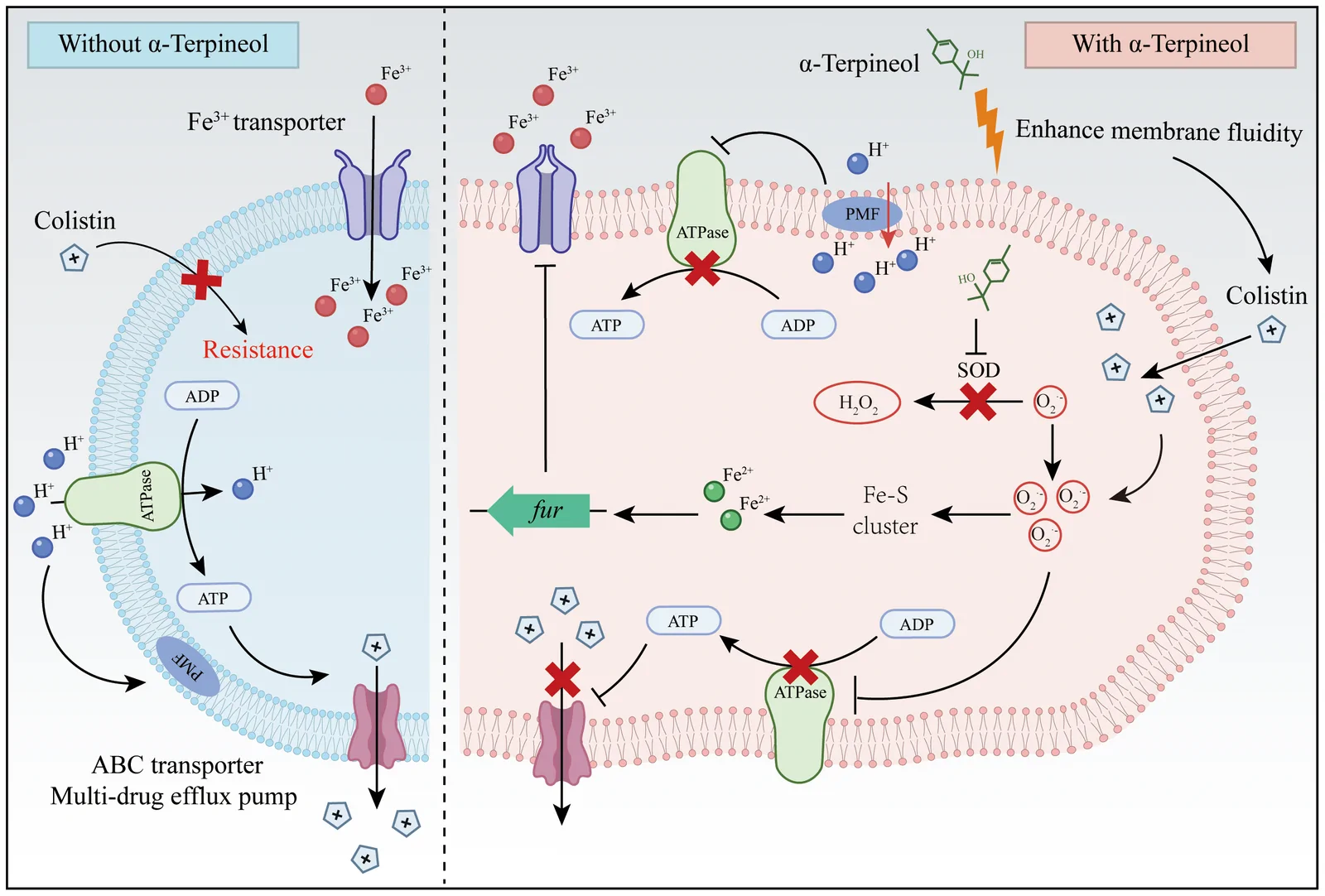
Schematic illustration of the potential mechanisms by which α-terpineol potentiates colistin activity against colistin-resistant MDR *E. coli*.

After penetrating the bacterial outer membrane, colistin can further damage the inner membrane, leading to O_2_^·–^ leakage. O_2_^·–^ attacks iron-sulfur clusters to release Fe^2+^ and is then converted by SOD into H_2_O_2_, which subsequently generates highly cytotoxic HO^·^ via the Fenton reaction, thereby damaging intracellular lipids, proteins, and DNA (Yeom et al., 2010). We measured the levels of O_2_^·–^ and found that α-terpineol assisted colistin in generating large amounts of O_2_^·–^ (**Figure 4A**). However, H_2_O_2_ and HO^·^ contents did not change significantly (**Figures 4B, C**), indicating that the accumulated O_2_^·–^ was not converted to H_2_O_2_ by SOD and that the highly cytotoxic HO^·^ burst did not occur. Moreover, α-terpineol significantly increased the intracellular Fe^2+^ level in a dose-dependent manner after colistin treatment (*P* < 0.001) (**Figure 4D**), without affecting the total iron content (**Supplementary Figure 4D**), suggesting that α-terpineol disrupted the Fe^2+^/Fe^3+^ balance during bacterial injury. We further investigated the function of SOD. Given that α-terpineol induces O_2_^·–^ accumulation in resistant *E. coli* and that Mn-SOD is the primary enzyme responsible for O_2_^·–^ scavenging under oxidative stress (Touati, 1989; Fee, 1991; Hopkin et al., 1992), we performed molecular docking to predict the interaction between α-terpineol and Mn-SOD (**Figure 4E**). The results showed that α-terpineol binds stably within the active pocket through two hydrogen bonds (Glu B170 and Tyr A34) in cooperation with multiple hydrophobic residues, with a binding affinity of –5.71 kcal/mol. MD simulations further confirmed the stability of this binding: analysis of the 100 ns trajectory revealed a stable complex (**Figures 4F–J**), with van der Waals and electrostatic interactions as the primary driving forces, and the ligand maintained a persistent hydrogen bond throughout the simulation. The average RMSD of the protein backbone was 0.20 ± 0.05 nm, indicating no substantial conformational change upon ligand binding (**Figure 4F**); the ligand RMSD was 0.13 ± 0.02 nm, reflecting high binding stability. RMSF analysis showed reduced fluctuations in residues near the binding pocket, suggesting enhanced local rigidity (**Figure 4G**). The Gibbs free energy landscape constructed from RMSD and Rg values revealed a low-energy basin corresponding to the stable bound state (**Figures 4H, I**; blue: low energy, red: high energy). The MM/GBSA-calculated total binding free energy (ΔG_total) was –21.34 ± 0.59 kcal/mol, indicating spontaneous and thermodynamically favorable complex formation. Based on these predictions, we measured SOD activity (**Figure 4J**) and observed that α-terpineol treatment significantly inhibited SOD activity in resistant *E. coli*. SOD plays a critical role in catalyzing the conversion of O_2_^·–^ to H_2_O_2_, and the decrease in its activity directly leads to impaired conversion of O_2_^·–^ to H_2_O_2_. To verify whether the synergistic effect was mediated by ROS, we performed functional validation by scavenging ROS. Given that O_2_^·–^ possesses reducing ability and can reduce Fe^3+^ to Fe^2+^ (Bradley et al., 2020a), supplementation of Fe^3+^ in the culture medium alleviated the oxidative damage caused by O_2_^·–^ accumulation, and the combined effect shifted from synergistic to indifferent (**Figure 4K**). Furthermore, vitamin C, as an oxidant scavenger, also alleviated oxidative stress, albeit with weaker efficacy than Fe^3+^, and the combined effect similarly shifted from synergistic to indifferent (**Figure 4K**). In summary, α-terpineol inhibits SOD activity, promoting the accumulation of colistin induced O_2_^·–^. The accumulated O_2_^·–^ subsequently attacks iron–sulfur clusters, directly impairing the function of iron–sulfur cluster dependent proteins (e.g., dehydrogenases), and disrupting the Fe^2+^/Fe^3+^ balance. Thus, α-terpineol achieves a dual effect: assisting colistin in inducing oxidative damage and compromising the bacterial antioxidant system (**Figure 5**).

### 3.4 α-Terpineol improves the therapeutic efficacy of colistin *in vivo*

**Figure 6:**
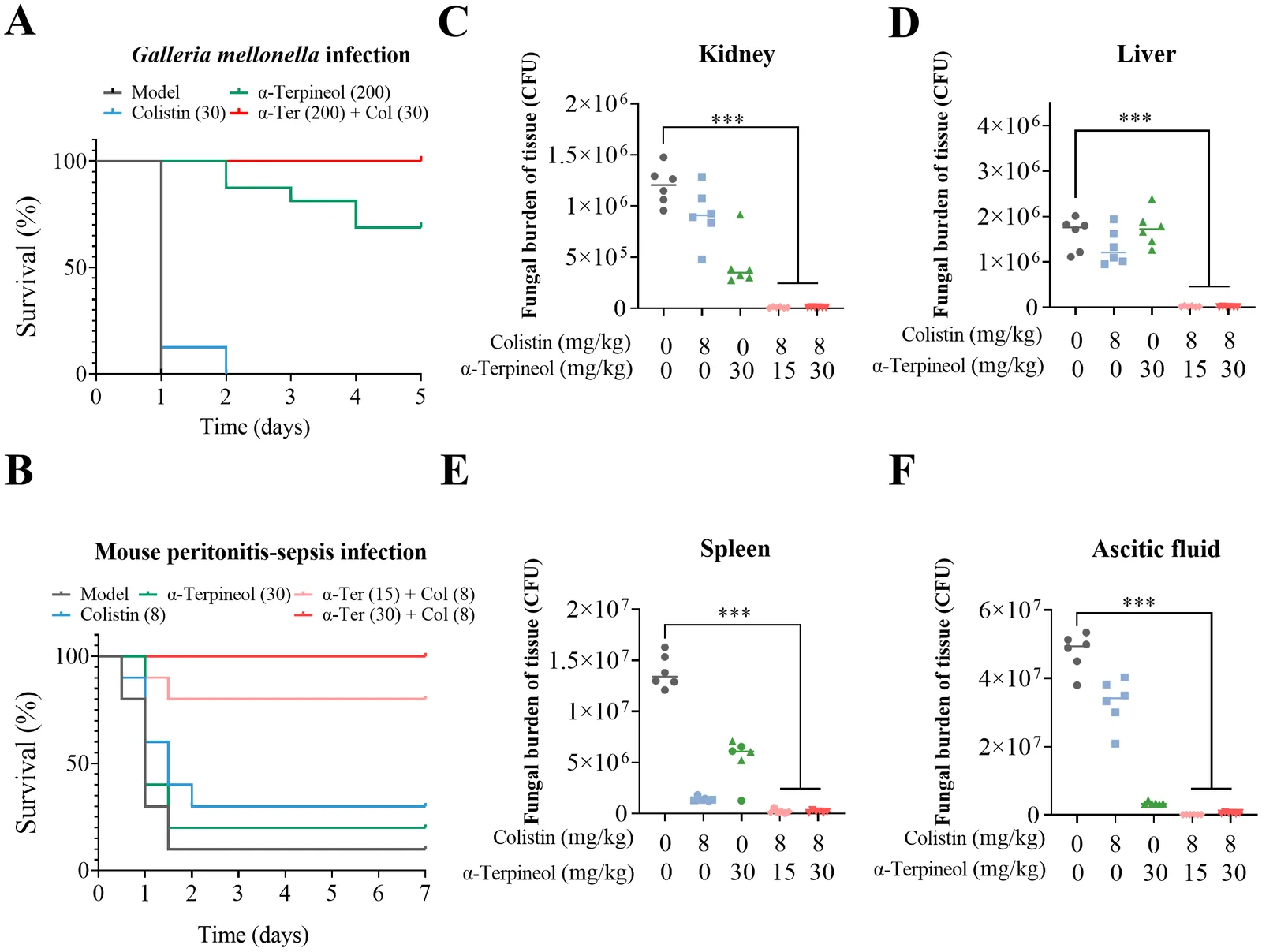
α-Terpineol restores colistin activity in two animal infection models. **(A)** The combination of colistin and α-terpineol (200 mg/kg) significantly improved the survival rate of *Galleria mellonella* larvae infected with colistin-resistant *E. coli* compared with colistin (30 mg/kg) monotherapy. **(B)** The combination of colistin (8 mg/kg) and α-terpineol (15 or 30 mg/kg) markedly increased the survival rate of mice infected with colistin-resistant MDR *E. coli* at 7 days post-infection in a dose-dependent manner. **(C–F)** The bacterial load in the internal organs (liver, kidney, and spleen) and ascitic fluid of mice was determined simultaneously. All experiments were performed with biological replicates and are presented as mean ± standard deviation. An unpaired one-way analysis of variance among multiple groups was used to calculate *P*-values (^*^P < 0.05, ^**^P < 0.01, ^***^P < 0.001 *vs* control).

Given the strong synergistic bactericidal activity of the combination of colistin and α-terpineol *in vitro*, we assessed its *in vivo* efficacy using two animal infection models. Experiments in the *G. mellonella* larval infection model showed that all larvae treated with saline or colistin alone died within 5 days post-infection. Treatment with α-terpineol alone resulted in a survival rate of 68.7% at 5 days. Remarkably, the combination therapy group achieved a 100% survival rate, which was significantly higher than that of either monotherapy group (**Figure 6A**). Similarly, in a mouse acute peritonitis model, neither colistin nor α-terpineol alone prevented lethal infection with colistin-resistant *E. coli*, with survival rates below 30%. In contrast, the combination therapy achieved survival rates of 80–100% (**Figure 6B**). Measurement of bacterial loads revealed that the combination therapy reduced bacterial counts in the liver, spleen, kidney, and ascitic fluid below the limit of detection (*P* < 0.001), whereas colistin or α-terpineol only partially reduced bacterial loads in some organs (**Figures 6C–F**). These data demonstrate that α-terpineol significantly restores the antibacterial activity of colistin *in vivo*.

## 4 Discussion

The spread of MDR Gram-negative bacteria threatens colistin, a last-line antibiotic, due to resistance mechanisms such as LPS modifications mediated by *mcr* genes (Peleg and Hooper, 2010; Mousavi et al., 2025; RoyChowdhury et al., 2025).Developing adjuvants to restore colistin activity is therefore a research priority (Li et al., 2024). α-Terpineol, a natural monoterpenoid alcohol derived from plants such as *Eucalyptus globulus* and *Pinus merkusii*, possesses multiple pharmacological properties such as antioxidant, antibacterial, antitumor, anti-inflammatory, and analgesic effects (Sabino et al., 2013; Ding et al., 2022; Masyita et al., 2022; Jin et al., 2024; Gopalsamy et al., 2025). α-Terpineol exerts its antibacterial activity primarily by disrupting bacterial membrane integrity and function (Xu et al., 2017). However, its potential as an antibiotic adjuvant against bacterial infections has been overlooked. This study demonstrates that α-terpineol significantly enhances the antibacterial efficacy of colistin against colistin-resistant MDR *E. coli*, and exhibits strong synergistic bactericidal activity. Notably, this combination did not further induce resistance and showed low cytotoxicity, consistent with the profile of an effective antibiotic adjuvant. Nevertheless, the current findings are limited to *mcr*-positive *E. coli*, and the generalizability of this synergy to other clinically relevant Gram-negative pathogens, remain to be determined.

Traditionally, colistin is known to exert antibacterial activity through membrane lysis—binding electrostatically to LPS in the outer membrane of Gram-negative bacteria, causing membrane expansion and structural disruption, followed by penetration of the inner membrane and phospholipid bilayer disturbance, ultimately leading to cell lysis (Dixon and Chopra, 1986; Bialvaei and Samadi Kafil, 2015; Yu et al., 2015). Recent studies indicate that colistin can also induce bacterial death via a hydroxyl radical (HO^·^) pathway, which involves ROS generation—including superoxide (O_2_^·–^), H_2_O_2_, and HO—upon inner membrane penetration (Sampson et al., 2012). In this pathway, colistin triggers O_2_^·–^ production, which is rapidly converted by SOD to H_2_O_2_ (Kohanski et al., 2007; Yeom et al., 2010). Both O_2_^·–^ and H_2_O_2_ can oxidatively attack iron–sulfur clusters, inactivating iron-sulfur-dependent enzymes (e.g., dehydrogenases) and releasing Fe^2+^ (Kohanski et al., 2007; Yeom et al., 2010). H_2_O_2_ can then oxidize Fe^2+^ via the Fenton reaction to generate highly toxic HO^·^ and ferric iron (Fe^3+^). The accumulation of HO^·^ induces oxidative damage to DNA, lipids, and proteins, resulting in rapid bacterial death (Dwyer et al., 2007; Yu et al., 2015). In this study, membrane lysis was not observed by SEM in resistant bacteria treated with the combination of α-terpineol and colistin; however, intracellular ROS levels were markedly elevated, and the synergistic effect could be antagonized by the ROS scavenger vitamin C (Dbouk et al., 2019). These findings were consistent with transcriptomic analysis, which linked the synergy to disrupted membrane function, redox imbalance, and impaired iron–sulphur metabolism. Further, membrane permeability assays show that α-terpineol induces bacterial shrinkage and increases inner/outer membrane permeability, thereby facilitating colistin penetration (Wenzel et al., 2018; Ginez et al., 2022). Although α-terpineol activated the colistin-induced HO^·^ pathway, HO^·^ levels did not increase significantly. Mechanistic investigations suggest that α-terpineol may suppress SOD activity, blocking the conversion of O_2_^·–^ to H_2_O_2_, leading to substantial O_2_^·–^ accumulation. Although O_2_^·–^ itself is not a potent oxidant, it can directly attack iron-sulfur clusters in the respiratory chain, releasing Fe^2+^ and causing damage (Wang et al., 2018). Exogenous Fe^3+^ can directly react with O_2_^·–^, converting it to O_2_, thereby mitigating damage (Bradley et al., 2020), which also enhanced bacterial tolerance to colistin. However, under SOD inhibition, the inability to convert O_2_^·–^ to H_2_O_2_ interrupts the Fenton reaction and leads to Fe^2+^ accumulation. High concentrations of Fe^2+^ activate the ferric uptake regulator gene (*Fur*), which subsequently represses exogenous iron uptake systems (Chiang and Schellhorn, 2012; Zhang et al., 2020). Consequently, protective Fe^3+^ cannot be absorbed, further exacerbating O_2_^·–^-mediated oxidative injury.

ATP serves as the energy foundation for critical physiological processes such as membrane potential maintenance, efflux pump function, and DNA repair in bacteria (Xu et al., 2017). The PMF, comprising the membrane potential (ΔΨ) and proton gradient (ΔpH), is generated through the inner membrane respiratory chain and drives ATP synthesis (Yang et al., 2023). In this study, α-terpineol reduced ΔΨ and dissipated the proton gradient, significantly disrupting PMF and thus inhibiting ATP production. Concurrently, O_2_^·–^ accumulation interfered with respiratory chain function via iron-sulfur cluster damage (Yu et al., 2015; Benarroch and Asally, 2020; Yang et al., 2023). The collapse of PMF and impaired ATP synthesis may collectively diminish the activity of energy-dependent efflux systems, such as ABC transporters and multidrug efflux pumps, potentially hindering drug extrusion and enhancing its intracellular accumulation and antibacterial efficacy (Wang et al., 2021; Cai et al., 2023). However, direct evidence, such as efflux pump activity assays or measurements of intracellular colistin accumulation, is still required to confirm whether the α-terpineol–colistin combination indeed inhibits efflux pump function and thereby increases intracellular colistin concentrations.

## 5 Conclusion

The synergistic mechanism of α-terpineol and colistin involves: (1) enhancing bacterial membrane fluidity to promote colistin penetration and elevate ROS levels; (2) suppression of SOD activity, disrupting the oxidative stress response and causing O_2_^·–^ accumulation; (3) O_2_^·–^-mediated attack on iron-sulfur clusters, disrupting iron homeostasis; and (4) iron-sulfur cluster inactivation and PMF collapse leading to ATP depletion and impaired efflux system function, preventing colistin extrusion. These interconnected mechanisms simultaneously subject bacteria to enhanced oxidative stress and energy failure, thereby potentiating colistin bactericidal activity against colistin-resistant strains. *In vivo* pharmacodynamic evaluations demonstrated that the combination of α-terpineol and colistin markedly improved survival rates in both *G. mellonella* infection and mouse acute peritonitis models. Therefore, we propose that α-terpineol is a promising colistin adjuvant that disrupts membrane homeostasis and antioxidant defenses, effectively restoring colistin activity against *mcr*-positive *E. coli*. This study provides novel mechanistic insights and a potential therapeutic strategy for combating colistin-resistant Gram-negative infections, although further investigations are warranted to definitively confirm the direct molecular targets, systemic safety, and clinical translational potential.

## Data Availability

The datasets presented in this study can be found in online repositories. The names of the repository/repositories and accession number(s) can be found in the article/**Supplementary Material**.
