# Supplementary material for: α-Terpineol reverses mcr-mediated colistin resistance and potentiates colistin’s antibacterial activity in multidrug-resistant Escherichia coli
Source: Front Cell Infect Microbiol. 2026 Aug 26;16:1908346. doi: 10.3389/fcimb.2026.1908346 (PMC13553474; doi:10.3389/fcimb.2026.1908346)
Supplement: Supplementary file 1 [file DataSheet1.pdf]

Supplementary Material

1 Supplementary Methods

1.1 Antimicrobial susceptibility testing

This method employs the standard broth microdilution technique to determine the minimum inhibitory concentrations (MICs). The highest concentration of each drug is set and then serially diluted two-fold in Mueller–Hinton broth (MHB). These drug dilutions were combined with a standardised bacterial suspension in a 96-well plate and co-cultured at 37 °C for 24 h. The final MIC results were interpreted for drug resistance according to the Clinical and Laboratory Standards Institute (CLSI 2024, USA) guidelines (CLSI 2024) and established literature to define the resistance profiles for different drug-bacterium combinations.

1.2 Real-time quantitative polymerase chain reaction (RT-qPCR)

A single *Escherichia coli* LN175 colony was grown overnight in MHB. The culture was then diluted 1:100 in fresh MHB and incubated for 6 h until it reached the exponential growth phase. Cells were subsequently treated with colistin (8 µg/mL) alone or in combination with α-terpineol (0.2 mg/mL) for 1 h. Total RNA was extracted using the Unizol Total RNA Extraction Reagent Plus (Genesand, Beijing, China) and quantified by measuring the absorbance ratio (A260/A280) in a TGem Ultra Spectrophotometer (Tiangen, Beijing, China). Reverse transcription was performed using the All-in-One 5× RT MasterMix (Applied Biological Materials, Richmond, Canada).

mRNA expression of the target genes was calculated using the $2^{-\Delta\Delta Ct}$ method relative to the control gene (16S rRNA) in *E. coli.* PCR amplification conditions were: initial denaturation at 95 °C for 180 s, followed by 40 cycles of 95 °C for 15 s and 60 °C for 60 s. RT-qPCR was performed using a LightCycler 96 instrument (Roche, Sweden). The primer pairs used for RT-qPCR analysis are listed in **Supplementary Table 1**.

1.2.1 α-Terpineol reduction assay

Ferric chloride ($FeCl_3$, 250 mmol/L) was incubated with α-terpineol to assess its ability to reduce ferric iron (Fe^{3+}). Subsequently, the concentration of ferrous ions was measured using a cellular ferrous ion colorimetric assay kit to assess changes in Fe^{2+} levels.

1.3 Safety assessment

The cytotoxicity of α-terpineol against mouse fibroblast L929 cells was evaluated using the Cell Counting Kit-8 (CCK-8) assay. L929 cells were seeded into 96-well plates at a density of 1×10^4 cells per well and cultured in Dulbecco's Modified Eagle medium supplemented with 5% foetal bovine serum for 12 h. Subsequently, the cells were treated with α-terpineol at final concentrations of 0, 0.4, 0.6, 0.8, 1.2, 1.6, 2.4, 3.2, 4.8, 6.4, and 9.6 mg/mL. After incubation at 37 °C in a 5% CO_2 cell culture incubator for 24 h, 10 µL of CCK-8 reagent was added to each well and incubated for 1 h. Absorbance (A) was measured at 450 nm using a multifunctional microplate reader. Cell viability rate was calculated as follows:

$$Cell\ viability\ rate\ (\%) = (A_{drug} - A_{blank})/(A_{control} - A_{blank})$$

The haemolytic activity of α-terpineol was evaluated using 4% rabbit red blood cells treated with α-terpineol (0.03–8 mg/mL) for 1 h. Triton X-100 (0.002–0.5%) served as the positive control, and physiological saline as the negative control. Following incubation, the absorbance (B) of the supernatant was measured at 576 nm and the haemolysis rate was calculated as follows:

$$Haemolysis\ ratio\ (\%) = (B_{drug} - B_{negative\ control})/(B_{positive\ control} - B_{negative\ control})$$

2 Supplementary Figures and Tables

2.1 Supplementary Figures

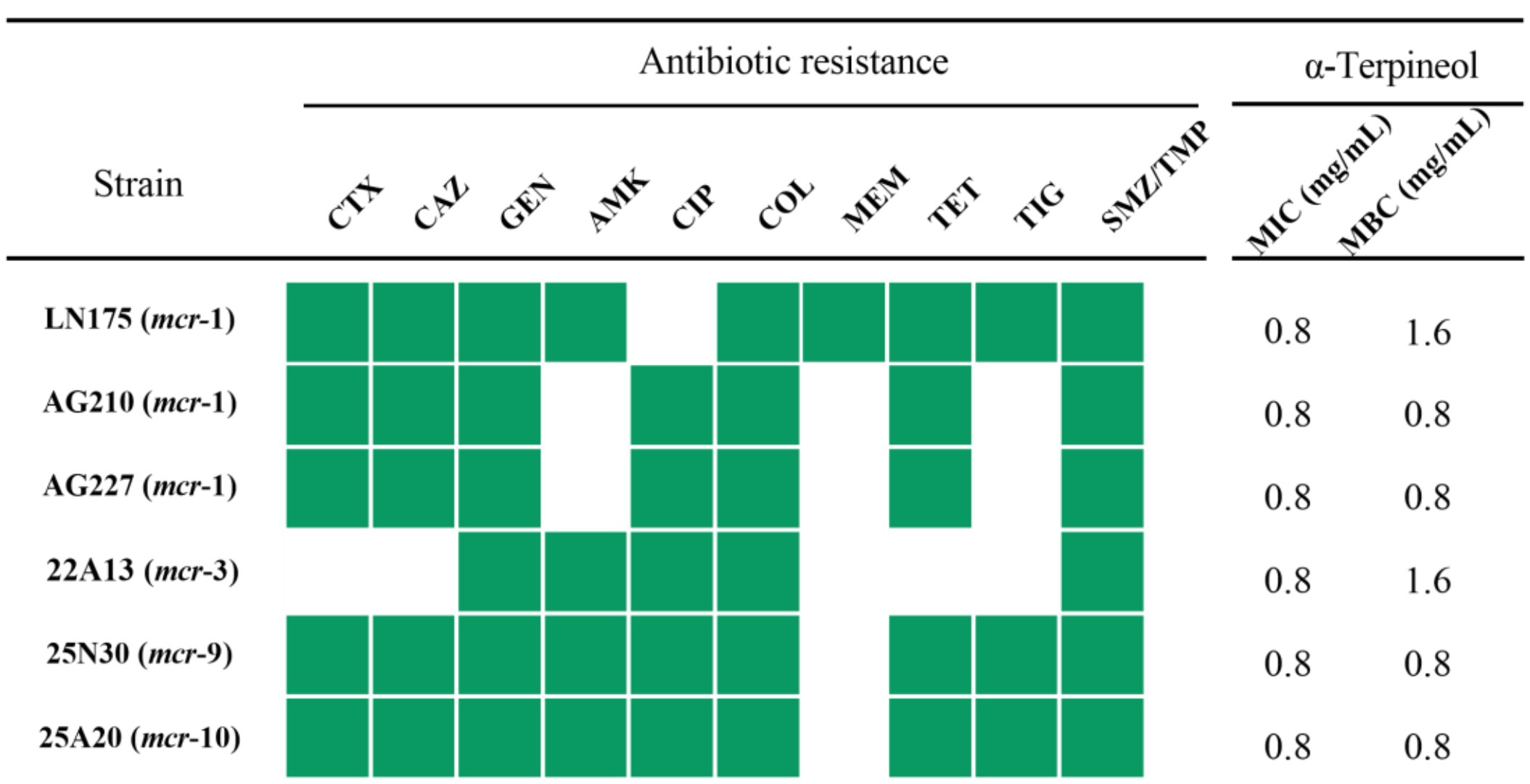

Supplementary Figure 1. Antimicrobial resistance profiles of the bacterial strains. Minimum inhibitory concentration (MIC) and minimum bactericidal concentration (MBC) values were determined according to Clinical and Laboratory Standards Institute (CLSI) 2024 standards. White

boxes indicate antibiotic sensitivity, green boxes indicate resistance, and the corresponding MIC and MBC values of α-terpineol are shown.

CTX, cefotaxime; CAZ, ceftazidime–avibactam; GEN, gentamicin; AMK, amikacin; CIP, ciprofloxacin; COL, colistin; MEM, meropenem; FLO, florfenicol; TET, tetracycline; SMZ/TMP, trimethoprim/sulfamethoxazole

Alt text: The heatmap presents the strain background information, using the presence or absence of color to indicate resistance: colored indicates resistance to the antibiotic, and colorless indicates sensitivity.

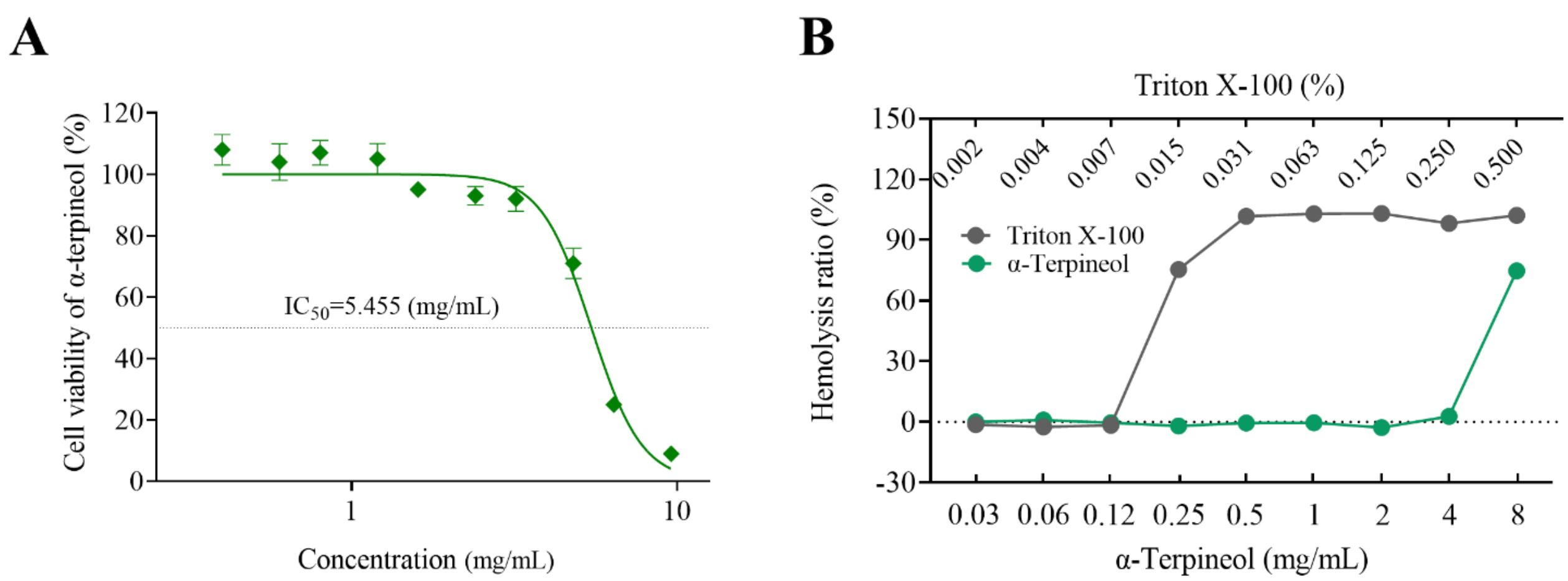

Supplementary Figure 2. Safety evaluation of α-terpineol. (A) The viability of L929 cells was assessed after treatment with the indicated concentrations of α-terpineol. (B) After treatment of rabbit red blood cells with varying concentrations of α-terpineol or Triton X-100, the degree of haemolysis was assessed. All experiments were performed with biological replicates, and results are presented as mean ± standard deviation.

Alt text: (A) is a line graph showing the change in cell viability with increasing α-terpineol concentration. (B) is a line graph showing hemolysis of red blood cells treated with α-terpineol and the comparator Triton X-100 as the concentration changes.

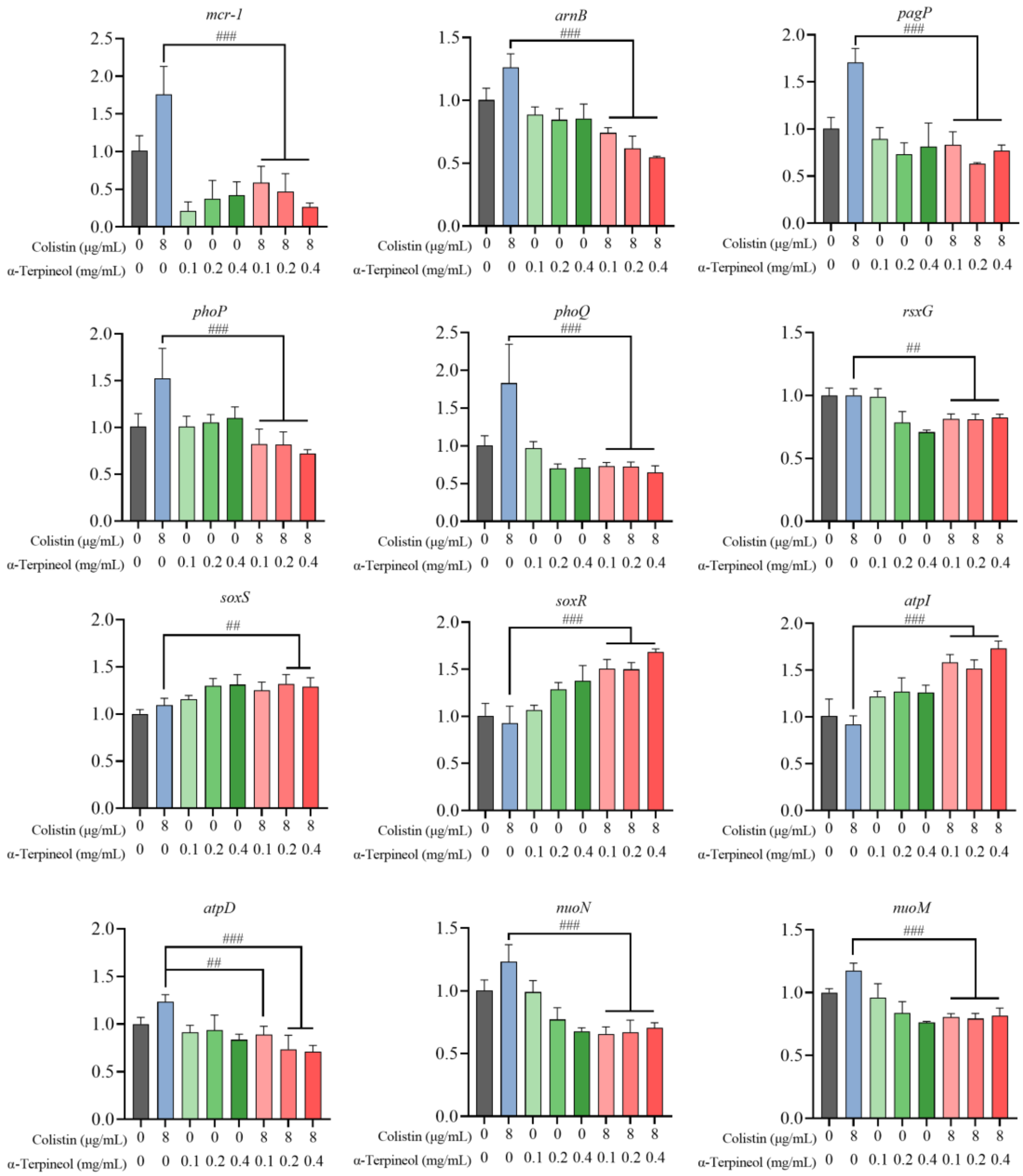

Supplementary Figure 3. Impact of α-terpineol on gene transcription was assessed using RT-qPCR. All experiments were performed with biological replicates, and results are presented as mean ± standard deviation. Unpaired one-way analysis of variance among multiple groups was used to calculate *P*-values ($^{\#}P < 0.05$, $^{\#\#}P < 0.01$, $^{\#\#\#}P < 0.001$ *vs.* colistin).

Alt text: The bar graph shows gene expression changes across different drug treatment groups, with statistical significance indicated.

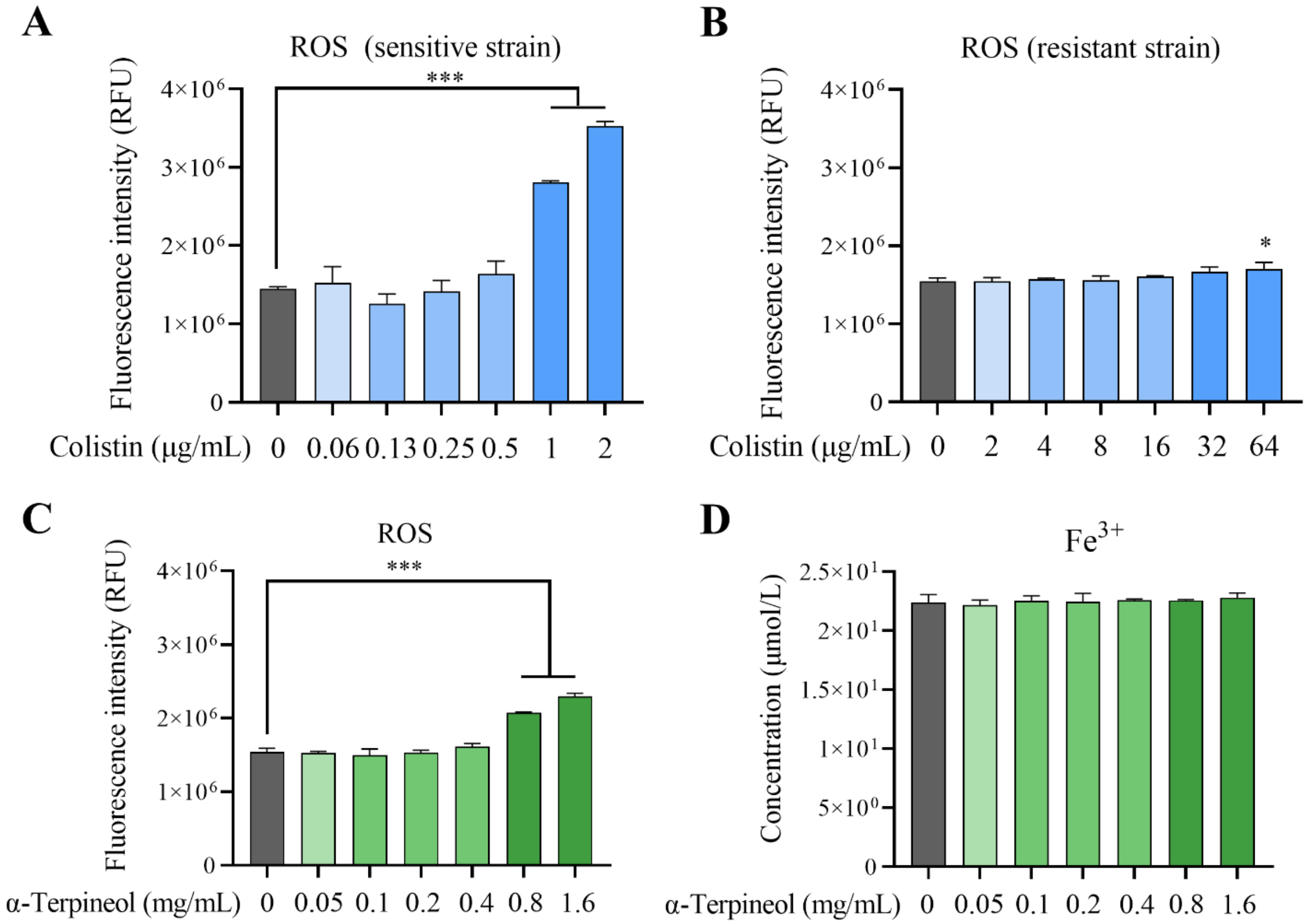

Supplementary Figure 4. Supplementary validation of oxidative damage induction. (A-B) The emergence of colistin resistance significantly increases the threshold concentration required to initiate the colistin-induced hydroxyl-radical death pathway in bacteria. (C) α-Terpineol at sub-bacteriostatic concentrations alone (≤0.4 mg/mL) had no direct effect on total reactive oxygen species (ROS) levels. (D) α-Terpineol does not directly transform Fe^{3+} into Fe^{2+}. All experiments were performed with biological replicates, and results are presented as mean ± standard deviation. Unpaired one-way analysis of variance among multiple groups was used to calculate *P*-values ($^{**}P<0.01$, $^{***}P<0.001$ *vs.* control).

Alt text： (A) to (D) are bar graphs, with statistical significance indicated. (A) and (B) compare the ROS production of colistin-resistant and colistin-susceptible bacteria under different colistin concentrations. (C) shows ROS production under different α-terpineol concentrations. (D) shows that α-terpineol alone had no effect on intracellular Fe^{2+} content.

2.2 Supplementary Table

Supplementary Table 2. Primers used for real-time quantitative polymerase chain reaction analysis

Gene	Primer	Sequence (5′–3′)
mcr-1	Forward	GACCAAGCCGAGACCAAGGA
	Reverse	CCACGATCAAGCCCAATCGG
arnB	Forward	GGCAAGGGCGATGAAGTGAT
	Reverse	GGCGTTGCACCCAACAAGGA
pagP	Forward	CAACGCGACAGGCCAAAACC
	Reverse	TGCTAACGCAGATGAGTGGA
phoP	Forward	ATGGTATGGCTTTCCCGCAGC
	Reverse	CAGGTTGATCTCTCTCGCCGT
phoQ	Forward	ATCCAGCACGTTGCCCATCAC
	Reverse	GCCCCACTGCTGGACAATCTC
rsxG	Forward	ACAATCCGGCGCGTTTTACC
	Reverse	GCACTGGGCGGTGAAGAAAG
soxS	Forward	TCGCTGGGAGTGCGATCAAA
	Reverse	ACCACCGAGCGTCCGATTTT
soxR	Forward	CGCAGCGGTGTGGCGGTATC
	Reverse	GCCGCTGTTACGGATACTGG
atpI	Forward	ACAGCCGGTGCCAGTATCTG
	Reverse	GGCGTTGGCGGTTTTAAAGG
atpD	Forward	ACCGACCATGTAGAACGCCT
	Reverse	GAAAGACACCATCCGTGGCT
nuoN	Forward	GGGATACCGGCCAGAGACAG

	Reverse	GCCCGGATGCTGATTCCCTG
nuoM	Forward	CCGGGACAACCTGGAAGCTG
	Reverse	CTGTCGCTGTTCTTTGCGGT
